# Repeatability of Inertial Measurement Units for Measuring Pelvic Mobility in Patients Undergoing Total Hip Arthroplasty

**DOI:** 10.3390/s23010377

**Published:** 2022-12-29

**Authors:** Sushanth Vayalapra, Xueyang Wang, Arham Qureshi, Abhinav Vepa, Usama Rahman, Arnab Palit, Mark A. Williams, Richard King, Mark T. Elliott

**Affiliations:** 1 Department of Trauma and Orthopaedics, University Hospitals Coventry & Warwickshire NHS Trust, Coventry CV2 2DX, UK; 2 WMG, University of Warwick, Coventry CV4 7AL, UK

**Keywords:** total hip arthroplasty, inertial measurement unit, pelvic tilt, wearables, reliability

## Abstract

Consideration of pelvic mobility when positioning implants for total hip arthroplasty (THA) has been shown to reduce the risk of complications such as dislocation, squeaking and excessive wear. We aim to test the repeatability of pelvic tilt measurements taken between three positions (standing, flexed-seated and step-up) by an inertial measurement unit (IMU) and hence, evaluate their reliability in screening for high pelvic mobility in patients undergoing THA. The repeated IMU measurements of pelvic tilt were analysed for consistency and compared with measures taken by x-ray analysis. Our study showed greater variation in measures taken by the IMU particularly in the flexed-seated position. The patient’s pelvic tilt in this position negatively correlated with their mid-back angle, suggesting the posture of the patient is a source of variation in the flexed-seated position if not kept consistent during assessments. IMUs were overall able to produce accurate and reliable measurements of pelvic tilt; however, protocols will need to be adjusted to factor in a patient’s mid-back angle when taking future readings.

## 1. Introduction

Pelvic mobility has been shown to be a key factor when considering the alignment of implants during total hip arthroplasty (THA) and reducing the risk of post-operative hip complications, such as dislocation, squeaking and excessive wear [1]. Hip dislocation is one of the most prevalent complications post THA and has a significant cost burden [2]. Historically, an increased risk of hip dislocation was thought to be related to the positioning of the acetabular cup and a “safe zone” of 40 ± 10° of inclination and 15 ± 10° anteversion for acetabular cup placement was identified [3]. Recent evidence suggests that placement of the acetabular cup within the safe zone does not always protect against dislocation, with the functional variation in pelvic tilt contributing to acetabular anteversion and hence, the increased risk of post-surgery complications [4,5,6]. To reduce this risk, patients with high functional pelvic tilt (i.e., >13 degrees anterior-posterior (AP) rotation between supine and flexed seated positions [5,7] should receive a personalised plan of the acetabular cup orientation, based on this pelvic range of motion, to minimise risk of post-surgery complications.

At present, measuring functional variation in pelvic tilt remains a challenge as assessments made during surgery are invasive and prolong time under anaesthetic [8,9,10]. Functional pre-operative radiographic imaging is a practical alternative which involves measuring pelvic tilt in a series of positions (e.g., sitting, standing) to optimise intra-operative acetabular cup component positioning and reduce dislocation rates [5,11,12]. However, this approach still has limitations in terms of increased exposure to radiation and the additional resources required to capture and analyse the measurements.

In recent years, there has been growing interest in the use of wearable inertial measurements units (IMUs) to provide objective measures of human motion for medical evaluation [13]. These devices have the benefit of being relatively low cost and portable as opposed to traditional means of clinical assessment [14]. IMUs can provide a series a repeated measurements which may give a more representative picture of a patient’s functional pelvic tilt rather than the single ‘snapshot’ provided by current radiographic methods. A recent study we conducted has shown the potential of using an IMU as a point-of-care screening tool to identify patients with high pelvic mobility who would benefit from more detailed surgical planning of implant positioning in the pre-operative setting [15]. The IMU and radiographic measurements in this previous study correlated strongly for flexed-seated (FS) position and moderately for step-up (SU) position. The overall accuracy of the IMU device was greater than 90% for identifying those individuals with high levels of pelvic mobility (defined as ≥13-degree change in pelvic rotation in either anterior or posterior directions). However, this previous study only compared a single sample of each of the IMU and radiograph measures for each patient. In this study we capitalise on the ability to rapidly capture multiple measurements of pelvic tilt within the same patient using the IMU device and sacral device evaluated by Wang et al. [15]. Compared to the single measures of pelvic tilt measured previously, here we can further evaluate the level of expected variability in a measurement through a patient repeating the same movements.

Repeatability is necessary to differentiate between measurement errors in the device and actual variation in a patient’s pelvic tilt when they perform the same movements and positions. The prior study we conducted highlighted potential measurement errors particularly when taking readings in the FS position [15]. This may be attributed to practical constraints such as the device hitting the seat base or back rest when in sitting position. It is important to investigate these potential sources of error so that adjustments can be made to mitigate these in future use. Although prior research has evaluated the repeatability of IMU devices in other contexts such as in gait measurement [16], no studies have examined the repeatability of these devices when measuring pelvic tilt in patients undergoing THA. 

In this study, we aim to assess the repeatability of pelvic tilt measurements taken by an IMU device placed in a bespoke sacral clamp in different functional hip positions.

### Aims

1. To compare repeated measures for change in pitch (AP tilt) between sitting and standing positions for the IMU device to assess displacement of the device during motion
2. To compare repeated measures for change in pitch with the IMU device with measurements taken by X-ray (XR) analysis
3. To determine the reliability of the IMU device by verifying consistency of repeated measures of pitch change
4. To identify if there is a correlation between mid-back angle and pitch in the flexed seated position when measurements are taken by the IMU device and XR

## 2. Materials and Methods

### 2.1. Pelvic Motion Tracking Device

**Figure 1:**
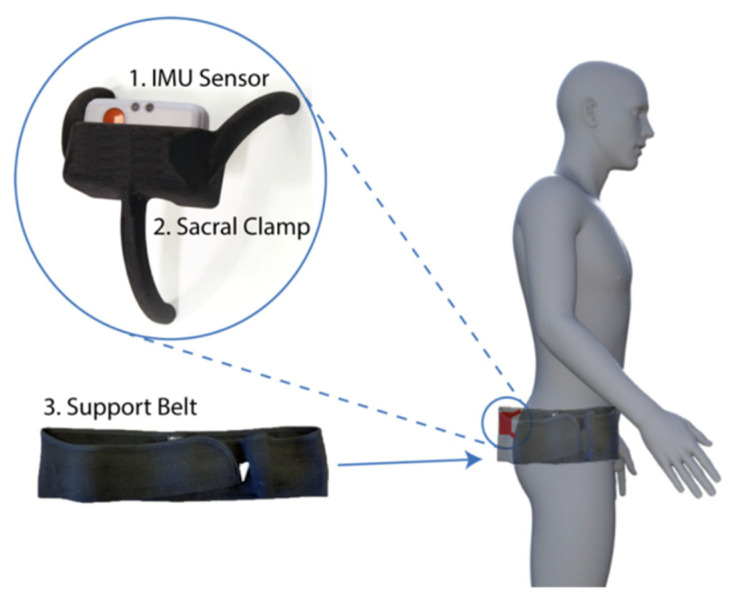
The three components of the device used to track pelvic motion: the inertial measurement unit (IMU) sensor, a bespoke sacral clamp and support belt. The device was attached across the sacrum and held in place by a pregnancy support belt. This belt was made from elasticated material allowing the individual to move freely, whilst holding the sacral clamp firmly in place. The sacrum had the least amount of skin/fat thickness between the device and the pelvic bone and hence it provided a feasible location to place the device for accurate pelvic tracking.

The device developed to track pelvic motion consisted of three main components namely the research grade IMU (Shimmer3; Shimmer, Dublin, Ireland), the sacral clamp and the support belt (Figure 1). The IMU (dimensions, 51 × 34 × 14 mm) housed three sensors: an accelerometer, gyroscopy and magnetometer. Only the data from the 3-axis accelerometer was used (sampling rate: 200 Hz; acceleration range: ±2 g) and transferred wirelessly to a host computer via Bluetooth connection. The IMU was calibrated, and the accuracy was assessed by tilting the device through a range of angles between −30 and +30 degrees using an industrial robot arm (KUKA KR10 R900, KUKA AG, Augsburg, Germany). Mean absolute error at ±30 degrees was recorded as 0.41 ± 0.32 degrees [15]. The IMU device was housed near the sacrum using a clamp as this area had the least amount of skin/fat between the pelvis and the device thereby allowing for more accurate measurement [15]. The support belt held the clamp securely around the waist whilst allowing the individual to move freely.

### 2.2. Participants

Patients were recruited through the University Hospitals Coventry and Warwickshire National Health Service (NHS) Trust as part of the Evaluation of X-ray, Acetabular Guides in THR (EXACT) trial [17]. Data from a total of n = 25 patients (mean age: 58 ± 7.1 years, female n = 12, mean height: 1.7 ± 0.1 m, mean weight: 84.9 ± 17.6 kg, mean BMI: 29.3 ± 4.3) was successfully collected as part of the study.

### 2.3. Ethics

The study formed part of the EXACT clinical trial and the protocol was approved by West Midlands-Solihull NHS Research Ethics Committee.

### 2.4. Procedure

An explanation on how the IMU device was to be fitted was provided to patients on arrival to the radiography room. A standing AP radiograph was taken prior to the IMU device being fitted. The IMU data recording was captured using a bespoke Matlab software script we developed in-house for this study (v2017a, Mathworks Corp., Natick, MA, USA), which linked to the device via Bluetooth connection.

**Figure 2:**
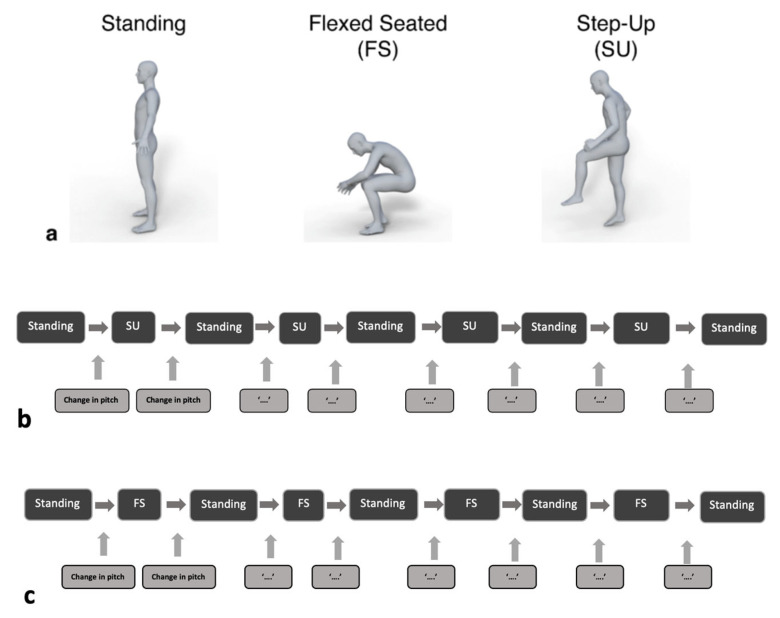
(**a**) The different positions assumed by patients during the procedure: standing, flexed-seated (FS) and step-up (SU). (**b**,**c**) Repeated measures were then taken in both FS and SU positions with return to standing position in between each measurement. Patients were asked to maintain each position for a minimum of five seconds, during which the IMU data and radiograph image were captured.

Patients were asked to adopt three positions: standing, flex-seated (FS) and step-up (SU) positions. These positions are common in daily activity and result in the highest angles of hip flexion in which pelvic tilt can be measured. The current standard practice for pre-surgical planning of implant position also involves adoption of these positions [17,18]. Initially, participants completed a series of these positions, with pelvic tilt recorded by both the IMU and radiograph (Figure 2a; [15]). Following this, a series of three repeated measurements were taken in between Standing and SU (Figure 2b) and then Standing and FS (Figure 2c) for the analysis of repeatability. The change in pitch was calculated with both the previous and following standing position, to help determine if the device had altered position between the movements.

In addition to measures of pelvic tilt, the mid-back angle was measured for the FS position, to determine the consistency of adopting this position—that is did participants lean forward by at the same angle for each repeated measure, and did this affect the pelvic tilt measure? This was measured using a digital inclinometer (Axminster Tools, Devon, UK) with a resolution of 0.1°. This was placed against the mid-back in the lower thoracic area, whilst patients adopted the FS position.

### 2.5. IMU and Radiographic Measures of Pelvic Tilt

**Figure 3:**
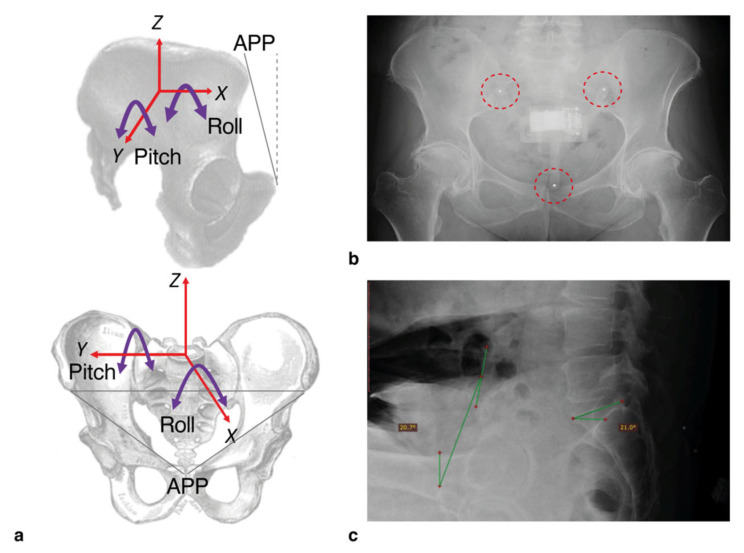
(**a**) The axes shown the image above demonstrate the measurements that can be taken from the IMU device. For this study, the only IMU measurement taken was pitch (i.e., tilt in the anterior-posterior direction, around the *Y*-axis). (**b**) The positioning of the device was checked by using 3 mm steel bearings inserted into the legs. The radiographic assessments were taken by measuring the angle of the anterior pelvic plane (defined as the plane between the anterior superior iliac spine and the pubic tubercle, relative to the vertical dashed line). (**c**) An example of this is shown.

Measurements of pitch (i.e., tilt in the AP direction) were calculated from the 3-axis accelerometer within the IMU device whilst participants assumed different functional hip positions (Figure 3a). The patients were requested to remain still for a period of 5 s during which data capture was conducted. The pitch and roll of the pelvis were calculated using two separate equations which were detailed in the previous study [14]. In this study, the focus was on pitch as the 2-dimensional radiographic images only provided this measure for comparison.

The protocol for measuring pelvic tilt from radiographs was as described in the current approach for measuring functional variation in hip position (Figure 3b,c) [19,20]. A lateral pelvic radiograph was used to calculate pelvic tilt in each of the three positions: standing, FS and SU. Change in pelvic tilt was recorded for FS and SU positions by calculating the difference between the tilt measured in these positions relative to the standing measure. An increase in anterior pelvic tilt was represented by a positive value.

### 2.6. Statistical Analysis

All data analyses were performed in SPSS for Mac version 27 (IBM, New York, NY, USA) and Microsoft Excel version 16 (Microsoft, Washington, DC, USA). A Shapiro–Wilk test was carried out to assess for the normal distribution of the data. For all the analyses we use the standing position as the reference tilt value and determine the change in pelvic tilt when participants adopt the FS and the SU positions. Standing position was captured immediately before and after each FS and SU position. The mean and standard deviation (SD) for change in pitch were calculated. A Paired two-sample *t*-test was used to compare to compare the difference in change in pitch. The statistical threshold was set a *p*-value of less than 0.05.

For each of the positions, an intraclass correlation coefficient (ICC) value was calculated to evaluate the reliability of the measurements. ICC estimates were calculated using SPSS based on a single measurement, absolute agreement, two-way mixed-effects model. The ICC values were interpreted using the reference points by Landis and Knoch [21] which indicate the following: 0.20 or less: mild; 0.21–0.40: fair; 0.41–0.60: moderate; 0.61–0.80: substantial; and 0.81 or greater: almost perfect.

Standard errors of the mean (SEMs) were calculated to measure the range of error for each position. The SEM was calculated for each position using the following formula: SEM = SD/√*n* where SD is the standard deviation and n is the number of samples [22]. The SEM was used to calculate the minimally detectable change (MDC) for each position by the following formula: MDC = SEM × 1.96 × √2 [22]. Pearson’s and spearman rho’s tests were used to assess for correlation between variables.

## 3. Results

### 3.1. Comparison of Step-Up and Flexed Seated Pitch Changes When Taken before and after Standing Position

There was no difference in the change in pitch when the patients moved to from Standing position to SU versus when they moved from SU to the Standing position (Paired T-test, Mean −1.93°, SD 2.89 vs. Mean −1.91°, SD 2.98°, *p* = 0.805). Similarly, a comparison in the difference in pitch between standing position both before and after FS position showed no statistical change (Paired T-test, Mean 7.63°, SD 16.49° vs. Mean 7.64°, SD 16.41°, *p* = 0.925).

### 3.2. Comparison of Changes in Pitch between Inertial Sensor and Radiograph Measurements

A comparison between the single radiograph and the mean of the repeated sensor pitch measurements for SU position showed no difference (Paired T-test, Mean −2.37°, SD 4.73° vs. Mean −1.93°, SD 2.89°, *p* = 0.612). However, the comparison between the single radiograph measure and the mean of the repeated sensor pitch measurements for the FS position was significantly different (Paired T-test, Mean −1.75°, SD 17.96° vs. 7.63°, SD 16.49°, *p* = 0.005).

### 3.3. Reliability Analysis of IMU Measurements Taken in Different Functional Hip Positions

The reliability analyses for both step-up and flexed-seated positions showed ICC values of greater than 0.94 and a MDC between 1 and 5 (Table 1).

### 3.4. Correlation between Mid-Back Angle and Pitch in Flexed Seated Position for Both IMU and XR Measurements

**Figure 4:**
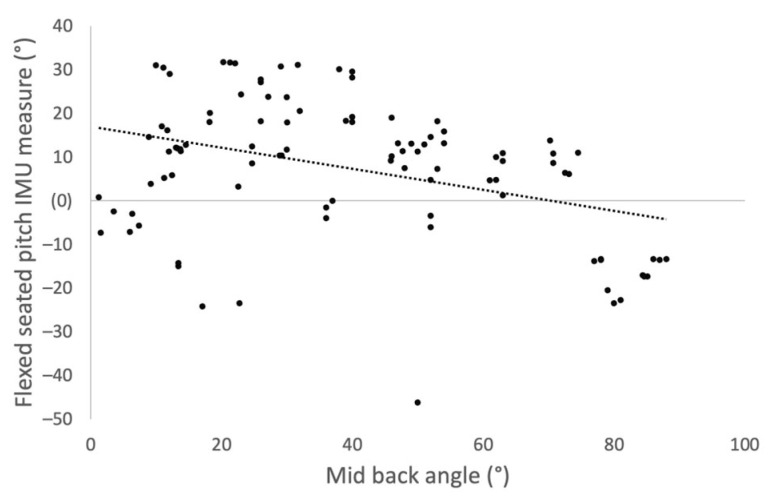
Scatter plot showing correlation between mid-back angle and change in pitch measured by the IMU device when the participants moved into the flexed seated position from standing.

**Figure 5:**
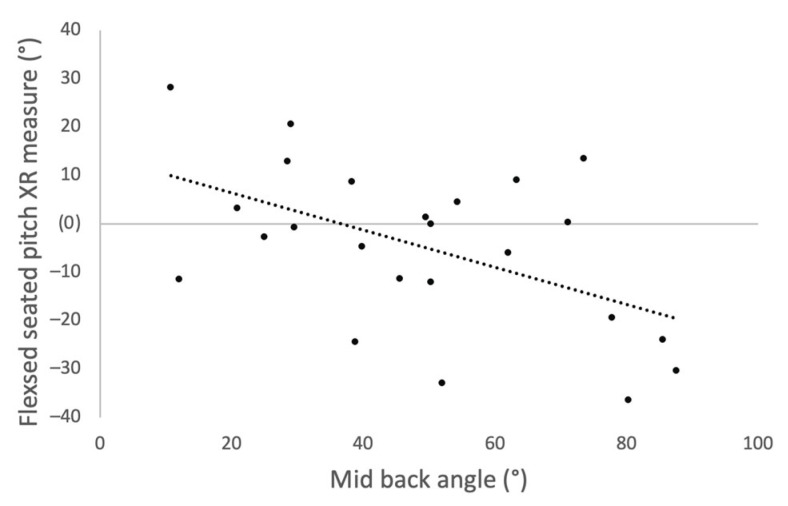
Scatter plot showing correlation between mid-back angle and change in pitch measured by XR when the participants moved into the flexed seated position from standing.

A negative correlation was identified between mid-back angle and the pitch measurements taken by the IMU device in the FS position (Figure 4, Spearman rho, R = −0.255, *p* = 0.012) and for measurements taken by XR (Figure 5, Spearman rho, R = −0.432, *p* = 0.035).

## 4. Discussion

This study expands on previous work which aimed to improve THA stability by promoting patient-specific tailoring of surgical implant positioning using data obtained through dynamic pre-operative assessment of hip biomechanics. This approach is more in line with modern schools of thought in which reliability of the Lewinnek Safe Zone has been contested [23,24].

A key step in the process of translating experimentation and innovation to clinical practice is assessing the reliability of the scientific method. Our previous study indicated that the new device could provide accurate readings and as such this study sought to assess the repeatability of these readings through iterations of measurements [15].

Repeated measures taken by the IMU device in FS and SU positions showed good reliability and repeatability with ICC values >0.94 for both. The prototype device therefore performed well when repeated measures were taken, considering the difficulty of tracking movements of the pelvis. An MDC of >4 degrees for FS compared to <2 degrees for SU suggests a greater degree of measurement error in the FS position. The acceptable limits of reliability for the clinical application of inertial sensors remains an area of debate. A prior study which evaluated the reliability of three-dimensional kinematic gait measurements suggested errors of between 2 and 5 degrees may be regarded as reasonable but the exact degree of acceptable measurement variation depends directly on the intended application [25]. Typical measures for changes in pelvic tilt can have a wide range (approx. ±30 degrees) and the threshold for high pelvic mobility is a change of ≥13 degrees in pelvic rotation in either anterior or posterior directions. Therefore, errors of <5 degrees from the device when used in the context of a screening tool may not be clinically significant as patients with high pelvic mobility will undergo further assessment with XR prior to operative intervention.

Our results further show that repeated measures of the IMU device taken before and after standing position were consistent for flexed-seated and step-up positions, suggesting that the sacral clamp remained in position during patient movement. Differences were noted between the repeated measures and those taken by XR interpretation in the flexed-seated position. This difference is likely due to variation in the patient’s mid-back angle when adopting the flexed-seated position. A negative correlation was found between the patient’s mid-back angle and the pitch of their pelvis in the flexed-seated position. Therefore, for a consistent measurement of pelvic tilt in the FS position, a more rigorous protocol is required that defines a specific mid-back angle for the patient to adopt.

Previous work evaluating the reliability of measurements by IMU devices in the context of measuring pelvic mobility showed comparable results. Bolink et al. compared IMU against motion capture (MOCAP) based measurements of pelvic range of motion during gait, sit-to-stand transfers and block step-up [26]. The waveforms that were captured for measuring pelvic motion during these three stages showed good agreement between IMU and MOCAP as ICC values were greater than 0.96. Buganè et al. compared the spatial orientation of the pelvis in three angles measured by an inertial sensor to that of a multiple TV camera stereophotogrammetric system and showed ICC values of between 0.88 and 0.95 [27].

A few limitations of this study should be acknowledged when interpreting the results. The repeated measures were taken on a limited cohort of 25 patients undergoing total hip arthroplasty. Given the small sample size, there may be some limitations to the generalisability of the results, although most of the patients were older and would match the common age demographic of patients with osteoarthritis. A further limitation is that measurement errors from the device are difficult to distinguish from functional variation in a patient’s pelvic tilt. An ideal study design would employ the comparison of repeated index test measurements to that of the reference standard. That is, each IMU measurement should be compared to that of an XR assessment to be able to distinguish variation in readings due to test error from potential natural variation in human hip movement. However, this was not a feasible study design because it would require participant exposure to unnecessary and unjustifiable excess radiation.

## 5. Conclusions

Measures of pelvic tilt taken by an IMU device positioned across the sacrum in a limited cohort of patients undergoing total hip arthroplasty show good repeatability and support its use as a screening tool for high pelvic mobility. The findings were consistent with those of previous studies which evaluate the use of IMU devices in tracking pelvic motion. Repeatability of the measurements could be improved with adjustment of data capture protocols to factor in a patient’s mid-back angle in the flexed-seated position. Further studies evaluating IMU measures of pelvic tilt on a larger, more diverse cohort of patients are needed prior to its use in the clinical setting.

## Figures and Tables

**Table 1:** Reliability analysis of IMU measurements in different positions.

Position	Mean (SD)	ICC	SEM	MDC
Step-up	−1.93 (2.89)	0.945	0.65	1.81
Flexed-seated	7.63 (16.49)	0.971	1.71	4.74

Abbreviations: SD (standard deviation); ICC (intra-class correlation coefficient; SEM (Standard Error of the Mean); MDC (Minimal Detectable Change).
